# Vancomycin-resistant *Staphylococcus aureus* endangers Egyptian dairy herds

**DOI:** 10.1038/s41598-024-81516-6

**Published:** 2024-12-23

**Authors:** Yasmine H. Tartor, Mohamed E. Enany, Noreen I. Ismail, Azza S. El-Demerdash, Nada H. Eidaroos, Reem M. Algendy, Yasser Mahmmod, Ibrahim Elsohaby

**Affiliations:** 1 Department of Microbiology, Faculty of Veterinary Medicine, Zagazig University, Zagazig, 44511 Egypt; 2 Department of Bacteriology, Immunology, and Mycology, Faculty of Veterinary Medicine, Suez Canal University, Ismailia, 41522 Egypt; 3 Veterinary Medicine Directorate, Sharkia, Egypt; 4 Agriculture Research Center (ARC), Animal Health Research Institute (AHRI), Zagazig, 44516 Egypt; 5 Department of Food Hygiene, Safety and Technology, Faculty of Veterinary Medicine, Zagazig University, Zagazig, 44511 Egypt; 6 Department of Veterinary Clinical Sciences, College of Veterinary Medicine, Long Island University, 720 Northern Boulevard, Brookville, NY 11548 USA; 7 Department of Infectious Diseases and Public Health, Jockey Club College of Veterinary Medicine and Life Sciences, City University of Hong Kong, Hong Kong, SAR China; 8 Centre for Applied One Health Research and Policy Advice (OHRP), City University of Hong Kong, Hong Kong, Hong Kong SAR China; 9 Department of Animal Medicine, Faculty of Veterinary Medicine, Zagazig University, Zagazig, 44511 Egypt

**Keywords:** VRSA, MRSA, Pandrug-resistant, Biofilm, *S. aureus*, Genotyping, Coagulase-negative staphylococci, Virulence, Mastitis, Microbiology, Diseases

## Abstract

The emergence of pandrug-resistant (PDR) and extensive drug-resistant (XDR) methicillin-resistant and vancomycin-resistant *Staphylococcus aureus* (MRSA and VRSA) isolates from bovine milk samples along with biofilm formation ability and harboring various virulence genes complicates the treatment of bovine mastitis and highlights the serious threat to public health. This study investigated for the first time the frequency, antimicrobial resistance profiles, biofilm-forming ability, virulence factors, *spa* and staphylococcal cassette chromosome *mec* (SCC*mec*) types of MRSA and VRSA isolated from clinical and subclinical bovine mastitis in Egypt. A total of 808 milk samples were collected from each quarter of 202 dairy animals, including 31 buffaloes and 171 cattle. The frequency of mastitis in the collected milk samples was 48.4% (60/124) in buffaloes and 29.2% (200/684) in cattle. A total of 65 *Staphylococcus* species isolates were recovered, including 27 coagulase-positive *S. aureus* (CoPS) isolates and 38 coagulase-negative staphylococci (CoNS). The CoNS included 27 mammaliicocci (20 *Mammaliicoccus lentus* and 7 *M. sciuri*) and 11 Non-aureus staphylococci (*S. lugdunensis*) isolates. All the CoPS isolates were *mec*A positive and resistant to 20–33 tested antimicrobials with multiple antibiotic resistance index ranging from 0.61 to 1. Three isolates were PDR, four were XDR, and 20 were multidrug resistant isolates. VRSA was detected in 85.2% of CoPS isolates with minimal inhibitory concentration (MIC) ranging from 64 to 1024 µg/mL. The *van*A gene was found in 60.8%, *van*B in 73.9%, and both genes in 43.5% of VRSA isolates. All the CoPS isolates exhibited biofilm formation ability, with 55.6% being strong, and 44.4% moderate biofilm producers, and harbored *ica*A (74.1%) and *ica*D (74.1%) biofilm-forming genes. All *S. aureus* isolates harbored both beta-haemolysin (*hlb*) and leucotoxin (*luk*MF) genes, while 44.4% were positive for toxic shock syndrome toxin (*tsst*) gene. Enterotoxin genes *sea*, *seb*, *sec*, *sed*, and *see* were found in 59.3%, 40.7%, 18.5%, 33.3%, and 14.8% of isolates, respectively. Additionally, 70.4% of the isolates had *spa* X-region gene, and exhibited eight different MRSA *spa* types (t127, t267, t037, t011, t843, t1081, t2663, and t1575), with *spa* t127 being the most common. Three SCC*mec* types (I, II and III) were identified, with SCC*mec* I being predominant, and were further classified into subtypes 1.1.1, 1.1.2, 1.n.1, and 4.1.1. The ability of MRSA and VRSA isolates to produce biofilms and resist antimicrobials highlights the serious threat these pathogens pose to bovine milk safety, animal welfare, and public health. Therefore, strict hygiene practices and antimicrobial surveillance are crucial to reduce the risk of MRSA and VRSA colonization and dissemination.

**Supplementary Information:**

The online version contains supplementary material available at 10.1038/s41598-024-81516-6.

## Introduction

Mastitis is one of the most prevalent diseases of bovines worldwide, causing significant economic losses by decreasing the quantity and quality of milk, increasing expenditures for drugs and veterinary services, and leading to death or early culling of affected animals^1^. *Staphylococcus aureus* (*S. aureus*) is one of the major causative agents of bovine mastitis, that causes clinical and subclinical infections in dairy cattle and buffalo and poses a potential health concern to humans^2^. Non-*aureus* staphylococci (NAS), considered minor mastitis pathogens, have variable effects on bovine udder health and milk production^3^. Recently, coagulase-negative staphylococci (CoNS) have been reclassified into NAS including *S. lugdunensis*, and mammaliicocci, including *Mammaliicoccus sciuri* and *M. lentus*^4^.

Antimicrobial therapy is crucial for mastitis control; however, *S. aureus* often develops resistance to multiple classes of antimicrobial agents due to the selective pressure of antimicrobials, which limiting the treatment options for clinicians and veterinarians^5^. The misuse of antibiotics such as using antibiotics without prescription, uncontrolled doses, or unnecessary application of drugs, has led to increased resistance of *S. aureus*^6^. The resistance to methicillin in methicillin-resistant *S. aureus* (MRSA) is a result of the expression of penicillin-binding protein 2a (PBP2a), which has a low affinity for *β*-lactam antibiotics. PBP2a is encoded by the *mec*A gene, which is located on the staphylococcal cassette chromosome *mec* (SCC*mec*)^7^. MRSA is multidrug resistant (MDR), not only resistant to *β*-lactam antibiotics but also to aminoglycosides, quinolones, and macrolides^8^.

Vancomycin, a glycopeptide antibiotic effective against Gram-positive bacteria, has been a cornerstone therapy for serious MRSA infections. However, excessive use has led to the emergence of vancomycin-resistant *S. aureus* (VRSA) strains^9^. Vancomycin resistance occurs through the horizontal transfer of a plasmid-borne transposon carrying the *van*A and/or *van*B genes from vancomycin-resistant *Enterococcus* to *S. aureus* crosswise the genus barrier that play a significant role in the development and dissemination of MDR^7^.

Besides antibiotic resistance, MRSA strains can form biofilms for survival and fitness. Strong adhesion, increased antimicrobial resistance, and decreased sanitizer efficacy are the factors that mediate this development^10^. Other virulence factors of *S. aureus* include leukotoxins, such as LukMF, the main toxin secreted in bovine mastitis and highly effective at killing bovine neutrophils^11^. Hemolysins (alpha, beta, gamma, delta) are major *S. aureus* virulence factors that facilitate invasion and evasion of the host immune response and play a crucial role in chronic mammary gland infections^12^. *S. aureus* can produce over 26 types of superantigens, such as staphylococcal enterotoxins (*SEs; SEA* to *-E*,* SEG* to *-J*, and *SER to -T*) and toxic shock syndrome toxin 1 (*tsst*−1). These toxins can overactivate specific T cells with uncontrolled release of proinflammatory cytokines, leading to various diseases in humans and animals, including mastitis in dairy cows^13^. Superantigens disrupt the normal immune response, impairing the body’s ability to effectively combat *S*. *aureus* infections. They can cause various diseases in humans, including food poisoning and toxic shock^14^. In bovine mastitis, they may facilitate *S. aureus* colonization and contribute to the establishment of persistent mastitis^15^.

Staphylococcal protein A (*spa*) is a key factor in *S. aureus* evasion of the immune system. By acting as a superantigen and disrupting the normal B-cell response, *spa* impairs the production of effective antibodies against *S. aureus*^16^. The *spa* gene, which codes for this protein, has a high degree of size variability^17^. The X-region of the *spa* gene contains repeating 24 bp repeats that vary throughout *S. aureus* strains, which is the source of this diversity in the gene. Therefore, *spa* typing can be employed as a molecular method to investigate genetic variation among *S. aureus* strains, thereby supporting the comparison of virulent phenotypes and epidemiological tracing of infection sources^18^. Moreover, SCC*mec* typing considered a useful tool for studying molecular epidemiology of MRSA isolates^18,19^.

There is limited data on the genetic diversity of VRSA and MRSA isolates causing clinical and subclinical mastitis^18^. Thus, the current study was designed to (i) investigate the frequency of MRSA, VRSA, and the Non-aureus staphylococci and mammaliicocci (NASM) in clinical and subclinical mastitis of cattle and buffaloes, (ii) determine the virulence determinants, biofilm formation ability of isolated MRSA and VRSA isolates, and (iii) identify the most common *spa* and SCC*mec* types of *S. aureus* isolates associated with bovine mastitis in Egypt.

## Materials and methods

### Animals and milk samples

A total of 808 milk samples were collected from each quarter of 202 lactating animals (171 cattle and 31 buffaloes) between October 2018 and April 2020. The samples were sourced from dairy farms in Sharkia and Dakahlia Governorates, Egypt, as well as from individual cases admitted to Animal Health Research Institute, Zagazig. The farms that were chosen employ extensive management strategies and hand milking. Four milk samples were collected from each animal, one from each quarter of the udder. Milk samples were aseptically placed into sterile screw-capped McCartney tubes (Thermo Fisher Scientific, Waltham, MA, USA), kept in an icebox and transferred to the microbiology laboratory for bacteriological examination. An informed consent was obtained from the owners of the farm to collect the bovine milk samples. All methods were carried out in accordance with ARRIVE guidelines (https://arriveguidelines.org) and regulations and approved by the institutional animal care and use committee at Faculty of Veterinary Medicine, Suez Canal University (Ethical approval no.: SZUC 201819).

### Somatic cell counting

The somatic cell count (SCC) was determined in fresh collected milk samples within 1 h after milking using a semi-quantitative California Mastitis Test (CMT) and a somatic cell counter (MT05, Slovakia). In CMT, 2 mL of milk sample was mixed with CMT reagent (Chimertech Private Limited, Chennai, India) and gently agitated in a four-well plastic paddle. The SCC is scored on a scale of 0 to 3, with scores of 2 or 3 considered based on changes in milk viscosity^20^.

The SCC was also measured automatically using MT05 somatic cell counter after mixing the milk sample (10 mL) with 5 mL of the 20% S4 reagent. SCC < 200,000 cells/mL of milk were considered normal, while ≥ 250,000 cells/mL were considered positive for subclinical mastitis, and those with over 1,000,000 cells/mL were classified as clinical mastitis^1^.

### Isolation and identification of *Staphylococcus* spp.

Milk samples were centrifuged at 3000 rpm for 5 min, the cream layer was discarded, and sediments were streaked onto Baird-Parker agar medium supplemented with an egg yolk–tellurite emulsion (Thermo Fisher Scientific Oxoid Ltd., Basingstoke, Hampshire, UK). Presumptive staphylococci colonies were subcultured onto mannitol salt agar medium (Thermo Fisher Scientific Oxoid Ltd., Basingstoke, Hampshire, UK), blood agar, and subjected to Gram staining, catalase, and coagulase tests^21^. The recovered *S. aureus* isolates were confirmed by amplification of *Sa0836* gene^22^ using primers listed in Table S1.

Coagulase-negative staphylococci (CoNS) isolates were further confirmed by amplification of 16S rRNA gene (Table S1)^23^. The VITEK^®^ technique (BioMérieux, Marcy l’Etoile, France) was employed for identification of CoNS isolates using ID-GpVITEK^®^ identification cards. For subsequent examination, the identified isolates were stored at −20 °C in brain heart infusion broth (BHI, Oxoid, Ltd., Basingstoke, Hampshire, UK) containing 30% glycerol.

### Antimicrobial susceptibility testing of ***S. aureus*** isolates

The Kirby-Bauer disc diffusion test was employed to determine the susceptibility of *S. aureus* isolates to 33 antimicrobial drugs representing 21 antibiotic groups commonly used in human and veterinary medicine, according to the Clinical and laboratory standards institute (CLSI) guidelines^24^. The tested antimicrobial agents including: amikacin (AK, 30 µg), amoxicillin-clavulanic acid (AMC, 20 µg/10 µg), ampicillin (AM, 10 µg), ampicillin/ sulbactam (SAM, 20 µg), bacitracin (B, 10 µg), ceftaroline (CPT, 30 µg), ceftazidime (CAZ, 10 µg), cefuroxime (CXM, 30 µg), cephradine (CE, 30 µg), ciprofloxacin (CIP, 5 µg), chloramphenicol (C, 30 µg), clindamycin (DA, 2 µg), daptomycin (DAP, 30 µg), erythromycin (E, 15 µg), doxycycline (DO, 30 µg), fosfomycin (FOS, 50 µg), fusidic acid (FA, 10 µg), gentamicin (CN, 10 µg), linzolid (LNZ, 30 µg), methicillin (ME, 5 µg), neitilmicin (NET, 10 µg), nitrofurantoin (F, 300 µg), norfloxacin (NOR, 10 µg), oxacillin (OX, 30 µg), penicillin (P, 10 µg), piperacillin + tazobactam (TZP, 10 µg), quinupristin/dalfopristin (QD, 15 µg), rifampin (RA, 5 µg), spiramycin (SP, 100 µg), tetracycline (TE, 30 µg), tigecycline (TGC, 10 µg), trimethoprim-sulfamethoxazole (SXT, 1.25 µg/23.75 µg), and vancomycin (VA, 30 µg). The rationale behind the choice of these antimicrobial drugs was to monitor the MDR, extensively drug-resistant (XDR), and pandrug-resistant (PDR) isolates^25^, aiming to address public health concerns.

The results were interpreted according to CLSI guidelines^24^. *S. aureus* ATCC 29737 reference strain was used as a control. The number of antimicrobial agents to which the isolate exhibited resistance was divided by the total number of antimicrobials tested to calculate the multiple antibiotic resistance (MAR) index^26^. If the isolate originated from a high-risk source with widespread antibiotic usage, the MAR index value is more than 0.2.

In accordance with the CLSI standards^24^, the isolates were further assessed using the broth microdilution test to determine the minimum inhibitory concentrations (MICs) of vancomycin (Sigma-Aldrich, USA). Vancomycin susceptible *S. aureus* (VSSA) isolates were those with MIC ≤ 2 µg/mL, whereas those with MIC of 4–8 µg /mL were classified as vancomycin intermediate *S. aureus* (VISA), and isolates with MIC > 16 µg/mL were considered vancomycin-resistant *S. aureus* (VRSA).

## Detection of biofilm formation ability and biofilm genes

Using 96-well flat-bottom polystyrene microtiter plates (Techno Plastic Products, Switzerland), the biofilm formation potential of *S. aureus* isolates (*n*= 27) was evaluated as previously described^27^. A sterile microtiter plate was inoculated with 200 µL of fresh cultures of each isolate in tryptic soy broth supplemented with 1% glucose (TSB, Thermo Fisher Scientific Oxoid Ltd., Basingstoke, Hampshire, UK) at 10^6^ CFU/mL. The plate was then incubated for 24 h at 37 °C. *S. aureus* ATCC 25923 and TSB with 1% glucose were employed as positive and negative controls, respectively. To get rid of non-adherent cells, the wells were aspirated and cleaned three times using 200 µL of phosphate buffer saline (PBS, pH 7.3). After a 15-min air-drying, the plates were drained. The biofilms underwent 30 min of staining with 150 µL of 0.1% crystal violet (Fluka AG, Buchs, Switzerland), followed by two PBS washes and air drying.

After resolving the stain that had bound to the cells with 150 µL of 95% ethanol for 45 min, the optical density (OD) was measured using an ELISA reader (Awareness Technologies stat fax 2100, CA, USA) at a wavelength of 570 nm.

Every isolate had a triplicate test, conducted three times. For both the tested isolates and the negative controls, average optical density (OD) values and standard deviations (SD) were determined.

The cut-off value of the OD (ODc), which is used to interpret the formation of biofilms, was computed as follows: ODc = average OD of negative control + (3 SD of negative control). The isolates were divided into four categories: weak (ODc < OD ≤ 2 ODc), moderate (2 ODc < OD ≤ 4 ODc), strong biofilm producers (4 ODc < OD), and non-producer (OD ≤ ODc).

Using primers listed in Table S1, biofilm-producing isolates were further tested for biofilm-related genes (*ica*A and *ica*D)^28,29^.

## Detection of methicillin- and Vancomycin- resistance genes

Using the QIAampDNA Mini Kit (Qiagen, GmbH, Germany) following the manufacturer’s instructions, genomic DNA was extracted from 24-h cultures of MRSA and VRSA isolates in brain heart infusion broth (BHI, Oxoid, Ltd., Basingstoke, Hampshire, UK). The NanoDropTM 1000 spectrophotometer (ThermoFisher Scientific, Waltham, MA, USA) was used to measure the quantity and purity of DNA. Using oligonucleotide primers (Table S1), the methicillin resistance gene (*mec*A) and the vancomycin resistance genes (*van*A and *van*B) were identified by PCR as previously described^29–32^.

PCR amplification was performed using 5 µL of the extracted DNA, 12.5 µL of 2X EmeraldAmpGT PCR master mix (Takara, Japan), 1 µL (20 pmol) of both forward and reverse primers (Metabion, Germany), and nuclease-free water to a final volume of 25 µL in a T3 Thermal cycler (BiometraGmbH, Göttingen, Germany). Methicillin- and VAN-susceptible *S. aureus* ATCC 29213, VAN-resistant *Enterococcus faecium* ATCC 51559 and *E. faecalis* ATCC 51299, and MRSA ATCC 33591 were used as positive and negative controls in each PCR amplification test. The 1.5% agarose gel containing 0.5 µg/mL ethidium bromide was used for electrophoresis analysis of the PCR-amplified products, and a gel documentation system (Alpha Innotech Corp., San Leandro, CA, USA) was used to visualise the results. To determine the molecular size, a 100 bp ladder (Cat. No. SM0243, Fermentas, USA) was employed.

## Detection of virulence genes

PCR amplification for staphylococcal enterotoxin genes (*sea*,* seb*,* sec*,* sed*, and *see*), toxic shock syndrome toxin (*tsst*), leukotoxin (*lukF* and *lukM*), and the hemolysin gene (*hlb*) (Table S1) was performed as previously described^33–36^. The amplification was carried out on a T3 Thermal cycler (Biometra, Germany). The PCR fragments were visualized using agarose gel electrophoresis and ethidium bromide staining.

### Typing of *S. aureus* isolates

#### DNA sequencing of *spa* gene

The X-region of the *spa* gene was amplified using primers 1095 F (5´-AGACGATCCTTCGGTGAGC-3´) and 1517R (5´-GCTTTTGCAATGTCATTTACTG-3´) (Midland Certified Reagent Company, oligos, USA)^37^. PCR products were purified using PureLink^®^ PCR Purification Kit (K3100-01, ThermoFisher, Waltham, MA, USA). Sequence reaction was prepared using the BigDye^®^ Terminator v3.1 Cycle Sequencing Kit (Applied Biosystems, USA). Multiple sequence alignment was conducted using ClustalW application in MEGA v7.2.5 software. *spa* types were determined with the Ridom StaphType software (Ridom GmbH, Wu¨rzburg, Germany) as previously described^37^. The sequence was submitted to GenBank using web tool *BankIt* of GenBank http://www.ncbi.nlm.nih.gov/WebSub/. Nucleotide sequences of *spa* gene were deposited in GenBank (accession nos: PP249546 - PP249564).

#### SCC*mec* typing

The isolates were further characterized by Staphylococcal cassette chromosome *mec* (SCC*mec*) typing using six multiplex PCRs to identify the *mec* gene complex, as described by Kondo et al.^38^

### Data analysis

Collected information and laboratory results were merged in excel sheet before being imported into STATA version 18 for Windows (Stata Corp., USA) and R software (version 4.2.0). The Chi-square (χ2) and Fisher exact test were used to assess the differences in proportion of mastitis cases between cattle and buffaloes and other factors. A significance level of < 0.05 was used to determine statistical significance. Correlations among resistance patterns, virulence and resistance genes and biofilm forming ability were evaluated by the Spearman’s rank correlation test and R package “corrplot”. The R package “Complex-Heatmap” was used to build heatmap^39^.

## Results

### Frequency of bovine mastitis

The overall frequency of mastitis in the examined animals was 32.2%, with 5.9% being clinical cases. The proportion of mastitis was 29.2% in cattle and 78.4% in buffaloes (Table 1). The frequency of mastitis was significantly higher (*P* < 0.05) in animals aged ≤ 4 years, backyard animals, cattle, and native breeds (Table 2).

**Table 1:** Frequency of clinical and subclinical mastitis in examined cattle and buffaloes.

Species	No. of animals	No. of milk samples	No. (%) of			Total mastitis cases
			CMT positive	SCC (> 200,000 cell/mL)		
				Subclinical	Clinical	
Cattle	171	684	50 (29.2)	41 (23.9)	9 (5.3)	50 (29.2)
Buffaloes	31	124	15 (48.4)	12 (38.7)	3 (9.7)	15 (48.4)
Total	202	808	65 (32.2)	53 (26.3)	12 (5.9)	65 (32.2)

No = number, CMT = California mastitis test, SCC = Somatic cell count.

**Table 2:** Number and percentage of mastitis cases in the examined cattle and buffaloes.

	No. of examined animals	No. (%) of mastitis cases (SCC > 200,000 cell/mL)	% (95% CI)	χ² *P*-value
**Year**				
2018	45	25 (55.6)	(40.0–70.4)	0.000
2019	91	35 (38.5)	(28.4–49.2)	
2020	66	5 (7.6)	(2.5–16.8)	
**Governorate**				
Sharkia	147	43 (29.3)	(22.1–37.3)	0.146
Dakahlia	55	22 (40.0)	(27.0–54.1)	
**Source**				
Backyard	90	38 (42.2)	(31.9–53.1)	0.006
Farm	112	27 (24.1)	(16.5–33.1)	
**Animal species**				
Cattle	171	50 (29.2)	(22.5–36.7)	0.036
Buffaloes	31	15 (48.4)	(30.2–66.9)	
**Breed**				
Native	62	29 (46.8)	(34.0–59.9)	0.007
Holstein	118	28 (23.7)	(16.4–32.4)	
Mixed	10	5 (50.0)	(18.7–81.3)	
Simmental	8	3 (37.5)	(8.5–75.5)	
Montbéliarde	4	0 (0.0)	0.0	
**Age**				
≤ 4 years	107	26 (24.3)	(16.5–33.5)	0.011
> 4 years	95	39 (41.1)	(31.1–51.6)	

The result is significant at *P* < 0.05.

### *Staphylococcus* spp. isolated from bovine mastitis

A total of 65 *Staphylococcus* spp. isolates were isolated from 65 mastitis cases, including 12 (18.5%) from clinical mastitis, and 53 (81.5%) from subclinical mastitis. Among these, 27 (41.5%) were identified as *S. aureus*, with nine (75%) from clinical mastitis and 18 (34%) from subclinical mastitis (Table 3). *S. aureus* isolates were *β*-hemolytic on blood agar, grew on Baird Parker agar media with the characteristic clear zones around black colonies, coagulase-positive and were further confirmed by PCR amplification of *Sa0836* gene that yielded 573 bp amplicons.

**Table 3:** Frequency of *Staphylococcus* spp. isolated from bovine mastitis.

Mastitis	No. of *Staphylococcus* isolates		No. (%) of *S. aureus* isolates		No. (%) of coagulase-negative *Staphylococcus* isolates	
	Cattle	Buffaloes	Cattle	Buffaloes	Cattle	Buffaloes
Clinical	9	3	7(77.8)	2(66.7)	2(22.2)	1 (33.3)
Subclinical	41	12	16 (39)	2(16.7)	25 (61)	10 (83.3)
Total	50	15	23 (46)	4(26.7)	27 (54)	11 (73.3)
	65		27 (41.5)		38 (58.5)	

No = number.

**Fig. 1:**
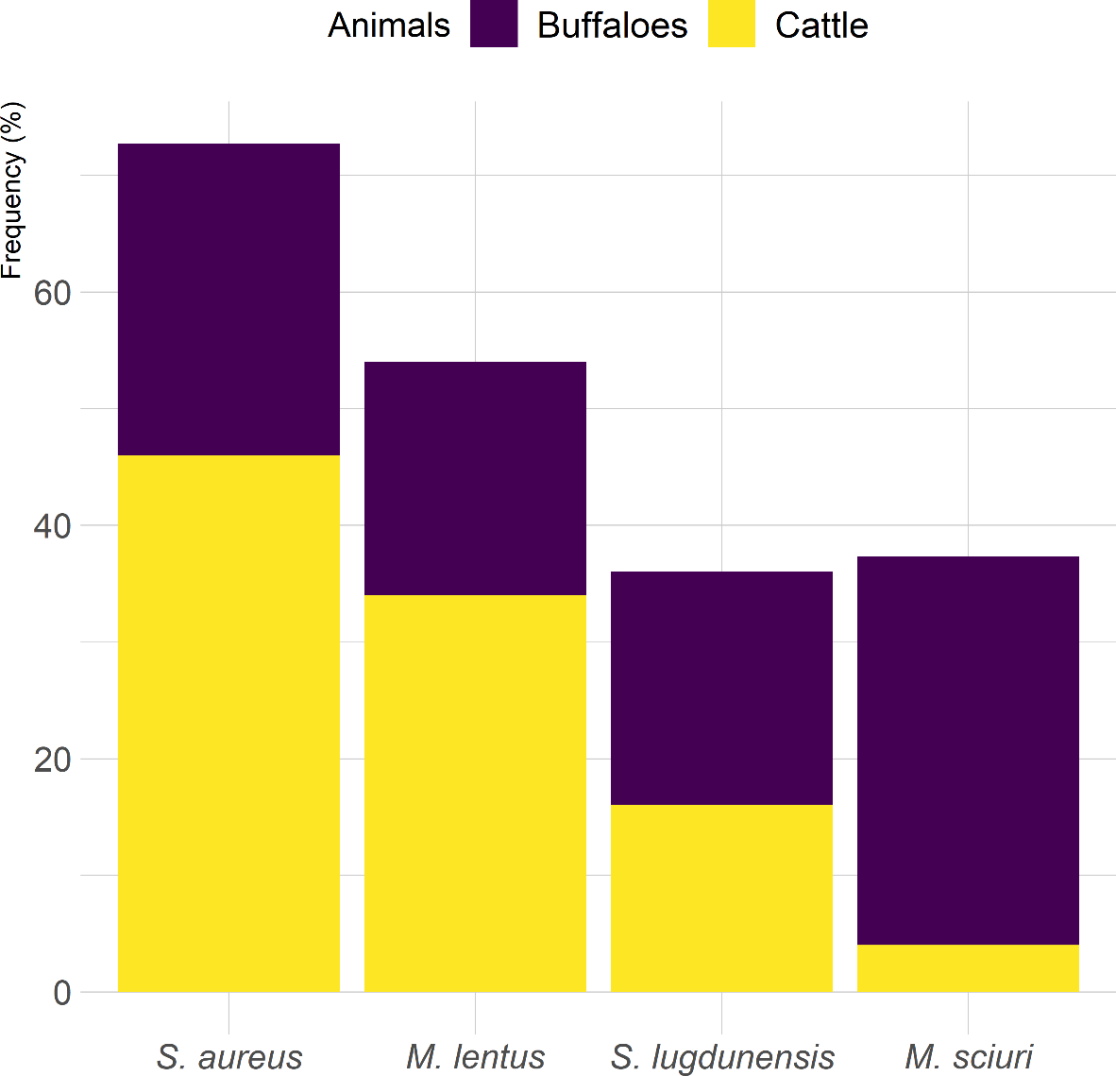
Frequency of *Staphylococcus* spp. and *Mammaliicoccus* spp. recovered from bovine mastitis cases.

The remaining 38 (58.5%) isolates were CoNS, with three (25%) from clinical mastitis, and 35 (66%) from subclinical mastitis (Table 3). These isolates were identified by VITEK 2 system as *Mammaliicoccus*, including *M. lentus* (*n* = 20), *M. sciuri* (*n* = 7), and *S. lugdunensis* (*n* = 11) (Fig. 1). These isolates were further confirmed by PCR amplification of 16 S rRNA gene that yielded 285 bp amplicons. Among the 20 *M. lentus* isolates, one (33.33%) was from clinical mastitis, and 19 (54.3%) from subclinical mastitis. Of the seven *M. sciuri* isolates, two (6%) were isolated from clinical mastitis and five (14.3%) from subclinical mastitis. All the 11 (31.4%) *M. lentus* isolates were recovered from subclinical mastitis.

### Antimicrobial susceptibility of *S. aureus* isolates

**Fig. 2:**
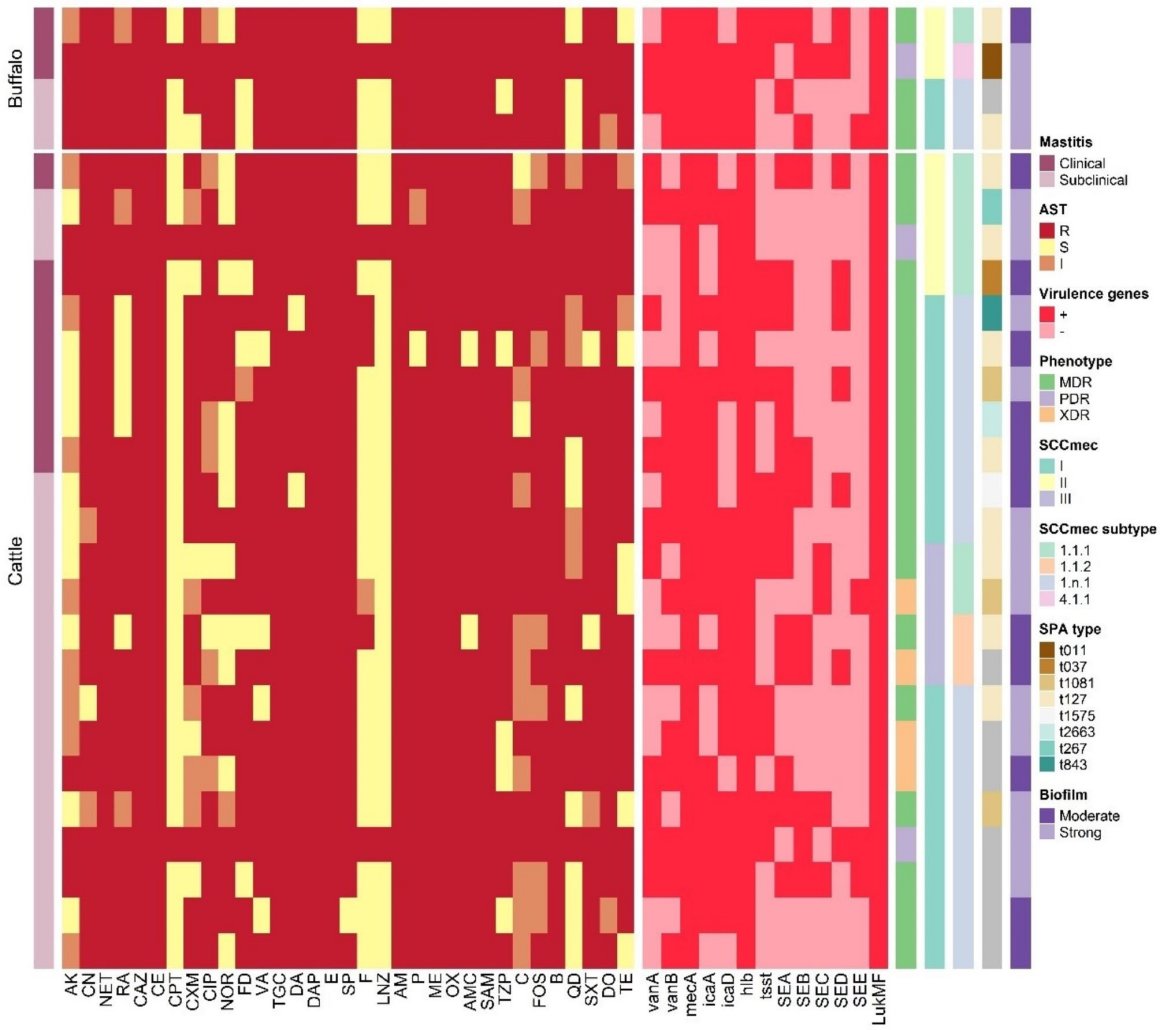
Heatmap representing antibiograms, biofilm formation ability, resistance, and virulence genes clustering of MRSA and VRSA isolates causing bovine mastitis.

According to the antibiogram profiles of the *S. aureus* isolates, 24 of the isolates had MAR index between 0.61 and 0.82 and were resistant to 20–27 antimicrobials. Twenty MDR isolates showed resistance to 19 antibiotic classes and four XDR isolates were resistant to 20 antibiotic classes with distinct antibiogram patterns. Three isolates were PDR and were resistant to all 33 tested antibiotics in 21 classes (MAR index = 1.0) (Tables 4 and Table 2S). Most MDR isolates were resistant to ampicillin, ampicillin/sulbactam, bacitracin, ceftazidime, cephradine, daptomycin, erythromycin, oxacillin, methicillin, neitilmicin, and tigecycline (100%), followed by spiramycin (96.3%), amoxicillin-clavulanic acid, clindamycin, doxycycline, and penicillin (92.6%). The resistance rates for amikacin (29.6%), nitrofurantoin (22.2%), and linzolid, ceftaroline (11.1%) were the lowest (Fig. 2).

**Table 4:** Antimicrobial susceptibility of *S*. *aureus* isolates causing bovine mastitis.

Class	Antimicrobial agent	Number (%) of *S. aureus* isolates (*n* = 27)		
		*R*	I	S
Aminoglycosides	AK	8 (29.6)	9 (33.3)	10 (37.1)
	CN	24 (88.9)	2 (7.4)	1 (3.7)
	NET	27 (100)	0.0	0.0
Ansamycin	RA	19 (70.4)	3 (11.1)	5 (18.5)
Cephalosporins	CAZ	27 (100)	0.0	0.0
	CE	27 (100)	0.0	0.0
	CPT	3 (11.1)	0.0	24 (88.9)
	CXM	17 (63.0)	5 (18.5)	5 (18.5)
Fluoroquinolones	CIP	19 (70.4)	6 (22.2)	2 (7.4)
	NOR	14 (51.9)	1 (3.7)	12 (44.4)
Fucidanes	FA	20 (74.1)	1 (3.7)	6 (22.2)
Glycopeptides	VA	23 (85.2)	0.0	4 (14.8)
Glycylcyclines	TGC	27 (100)	0.0	0.0
Lincosamides	DA	25 (92.6)	0.0	2 (7.4)
Lipopeptides	DAP	27 (100)	0.0	0.0
Macrolides	E	27 (100)	0.0	0.0
	SP	26 (96.3)	0.0	1 (3.7)
Nitrofurans	F	6 (22.2)	1 (3.7)	20 (74.1)
Oxazolidinones	LNZ	3 (11.1)	0.0	24 (88.9)
Penicillinase – labile Penicillins	AM	27 (100)	0.0	0.0
	P	25 (92.6)	1 (3.7)	1 (3.7)
Penicillinase - stable Penicillins	ME	27 (100)	0.0	0.0
	OX	27 (100)	0.0	0.0
Penicillin + β lactamase Inhibitors	AMC	25 (92.6)	0.0	2 (7.4)
	SAM	27 (100)	0.0	0.0
	TZP	22 (81.5)	0.0	5 (18.5)
Phenicols	C	15 (55.6)	10 (37.0)	2 (7.4)
Phosphonic acid	FOS	21 (77.8)	6 (22.2)	0.0
Polypeptides	B	27 (100)	0.0	0.0
Streptogramins	QD	12 (44.5)	5 (18.5)	10 (37.0)
Sulfonamides	SXT	24 (88.9)	1 (3.7)	2 (7.4)
Tetracyclines	DO	25 (92.6)	2 (7.4)	0.0
	TE	19 (70.4)	2 (7.4)	6 (22.2)

n, number of isolates; R, resistant; I, intermediate; S, sensitive.

AK, amikacin; AM, ampicillin; AMC, amoxicillin/clauvalanic acid; B, bacitracin; C, chloroamphenicol; CAZ, ceftazidime; CE, cephradine; CIP, ciprofloxacin; CN, gentamycin; CPT, ceftarolline; CXM, cefuroxime; DA, clindamycin; DAP, daptomycin; DO, doxycycline; E, erythromycin; F, nitrofurantoin; FD, fusidic acid; FOS, fosfomycin; LNZ, linezolid; ME, methicillin; NET, neitilmicin; NOR, norfloxacin; OX, oxicillin; P, penicillin; QD, quinopristin/ dalfopristin; RA, rifampin; SAM, ampicillin + sulbctam; SP, spiramycin; SXT, trimethoprim/Sulfamethoxazole; TE, tetracycline; TGC, tigecycline; TZP, pipracillin + tazobactam; VA, vancomycin.

Twenty seven isolates were MRSA. Of them, 23 (85.2%) isolates had resistance to vancomycin (VRSA), with MIC values ranging from 64 to 1024 µg/mL (Table 4 and Table 2S).

### The ability of MRSA and VRSA isolates to produce biofilms

All 27 MRSA isolates (100%) were biofilm producers, with 15 (55.6%) classified as strong and 12 (44.4%) as moderate biofilm producers (Fig. 2 and Table 2S). Additionally, all 23 VRSA isolates were biofilm producers, with 14 being strong and 9 moderate biofilm producers. Both *icaA* and *icaD* biofilm-producing genes were detected in 51.9% (14/27) of the isolates, while one isolate had neither gene. The *icaA* and *icaD* genes were each found in 74% of the isolates.

### Resistance and virulence genes in MRSA and VRSA isolates

All 27 MRSA isolates harbored the *mec*A gene. The resistance genes *van*A, *van*B, and both were found in 60.8%, 73.9%, and 43.5% of the 23 VRSA isolates, respectively.

Twelve out of 27 MRSA isolates (44.4%) harbored *tsst* gene, while the *hlb* (beta-haemolysin) and *luk*MF (leukotoxins) genes were detected in all isolates (100%). The enterotoxin genes *sea*, *seb*, *sec*, *sed*, and *see* were detected in 16 (59.3%), 11 (40.7%), 5 (18.5%), 9 (33.3%), and 4 (14.8%) of the 27 MRSA isolates, respectively (Table 2S and Fig. 2).

### Correlation between phenotypic and genotypic traits of *S. aureus* isolates

**Fig. 3:**
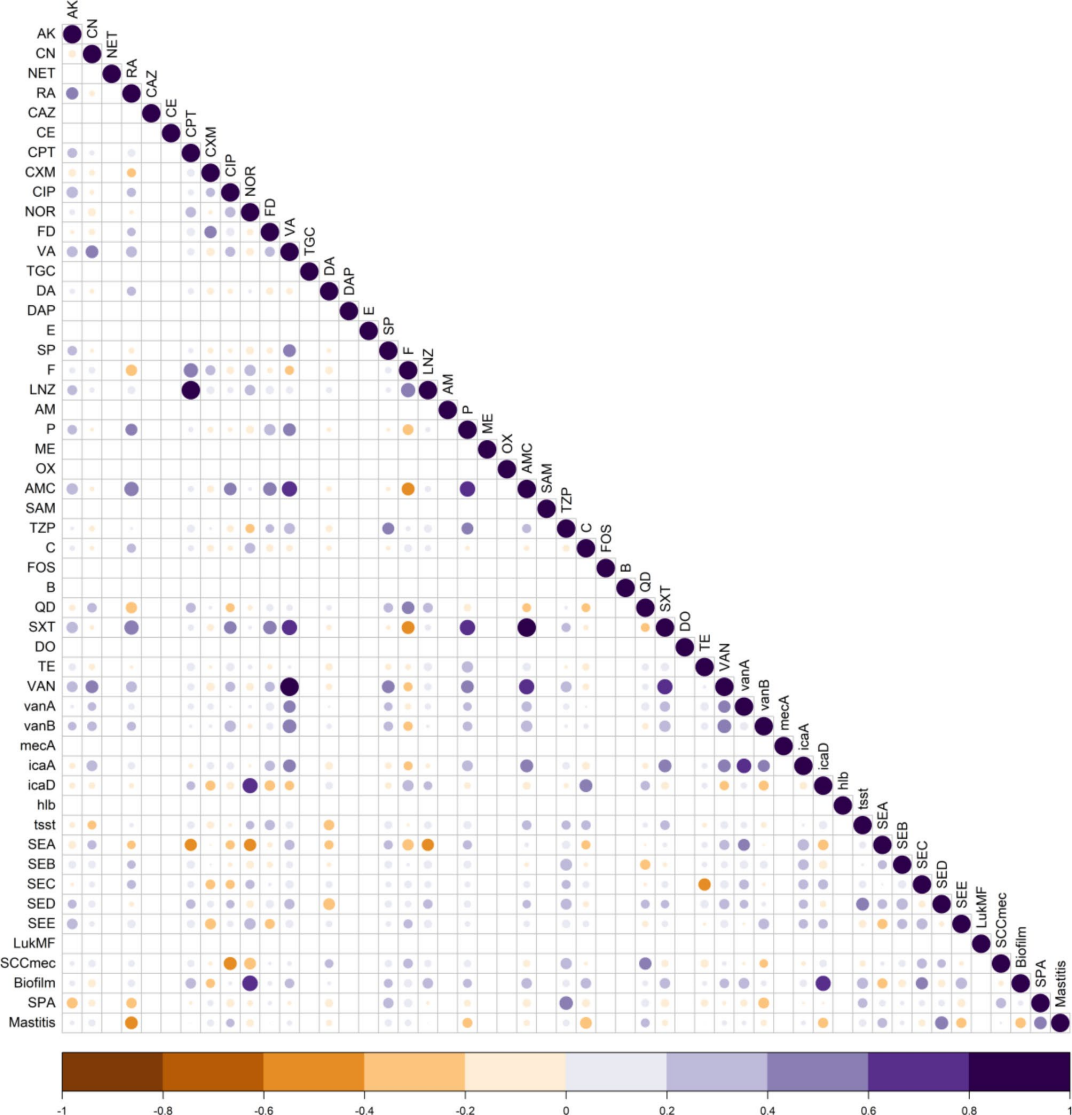
Correlation between resistance to antimicrobials, resistance genes, virulence genes, biofilm formation ability, and typing of MRSA and VRSA isolates.

Antimicrobial resistance has been correlated in different ways with the capacity of MRSA and VRSA isolates to form biofilms and to harbor virulence genes (Table 2S). Correlation matrix analysis was used to determine the relationships between phenotypic and genotypic traits, virulence and biofilm-forming abilities of the isolates (Fig. 3). Significant positive (*P* < 0.05) correlations were identified between biofilm formation and the presence of *ica*D gene and resistance to norfloxacin. Similarly, a positive correlation was observed between the presence of *van*A and *ica*A genes. Additionally, resistance to antibiotics such as vancomycin, linzolid, and ceftaroline showed significant positive correlations. Moreover, clinical mastitis was positively correlated with the presence of the *spa* gene and the virulence gene *sed*. Conversely, few significant negative correlations were observed between the presence of SCC*mec* and ciprofloxacin, and between virulence gene *sea* and ceftaroline, and norfloxacin and linzolid. These correlations indicate the co-occurrence of resistance and the presence of MDR, XDR, and PDR isolates.

### *spa* and SCC*mec* types

Nineteen isolates (70.4%) harbored the *spa* X-region gene. Based on *spa* typing, eight different *spa* types (t127, t267, t037, t011, t843, t1081, t2663, and t1575) were identified, with *spa* t127 being the predominant type (Table 5 and Table 2S).

**Table 5:** *spa* types of MRSA and VRSA isolates recovered from bovine clinical and subclinical mastitis.

*spa* type	*N*	No. (%) of *spa* types in					
		Farm	Backyard	Cattle	Buffalo	Clinical	Subclinical
t127	10	5 (50)	5 (50)	8 (80)	2 (20)	4 (40)	6 (60)
t267	1	0	1 (100)	1 (100)	0	0	1 (100)
t037	1	0	1 (100)	1 (100)	0	1 (100)	0
t011	1	0	1 (100)	0	1 (100)	1 (100)	0
t843	1	0	1 (100)	1 (100)	0	1 (100)	0
t1081	3	2 (66.7)	1 (33.3)	3 (100)	0	1 (33.3)	2 (66.7)
t2663	1	1 (100)	0	1 (100)	0	1 (100)	0
t1575	1	1 (100)	0	1 (100)	0	0	1 (100)
Total	19	9 (47.4)	10 (52.6)	16 (84.2)	3 (15.8)	9 (47.4)	10 (52.6)

Table 6 shows that three SCC*mec* types (I, II and III) were identified to detect the essential genetic components of *mec*A, with SCC*mec* type I being the predominant type (*n* = 17), and were further classified into subtypes (1.1.1, 1.1.2, 1.n.1, and 4.1.1). SCC*mec* type II (1.1.1 and 4.1.1) and type III (1.1.1 and 1.1.2) were found in six and four isolates, respectively.

**Table 6:** SCC*mec* types of MRSA and VRSA isolates recovered from bovine clinical and subclinical mastitis.

SCC*mec*		*N*	No. (%) of SCC*mec* types in					
Type	Subtype		Farm	Backyard	Cattle	Buffalo	Clinical	Subclinical
I	1.n.1	17	9 (52.9)	8 (47.1)	15 (88.2)	2 (11.8)	5 (29.4)	12 (70.6)
II	1.1.1	5	0	5 (100)	4 (80)	1 (20)	3 (60)	2 (40)
	4.1.1	1	0	1 (100)	0	1 (100)	1 (100)	0
III	1.1.1	2	2 (100)	0	2 (100)	0	0	2 (100)
	1.1.2	2	2 (100)	0	2 (100)	0	0	2 (100)
Total		27	13 (48.1)	14 (51.9)	23 (85.2)	4 (14.8)	9 (33.3)	18 (66.7)

## Discussion

Bovine mastitis is a disease that seriously impairs cattle productivity and costs the livestock business money globally, including Egypt^40^. In this study, we aimed to provide detailed insights into the frequency of MRSA, VRSA, and NASM in clinical and subclinical bovine mastitis in Egypt, along with genotyping, resistance traits, and virulence profiles of MRSA and VRSA isolates. The inclusion of subclinical mastitis cases is important because this milk is often consumed by farmers or sold for human consumption without pasteurization.

In the milk samples taken from each quarter, the overall frequency of mastitis was 32.2%; this included 48.4% in buffaloes and 29.2% in cattle. This was further classified into 9.7% clinical, and 38.7% subclinical mastitis in buffaloes, and 5.3% clinical, and 23.9% subclinical mastitis in cattle. These results are similar to the 39.9% reported in Ethiopia^41^, and 44% in buffaloes and 52.1% in cattle reported in Egypt^42^. However, higher frequencies were also reported in Ethiopia (63.02%^43^), and Uganda (86.2%^44^).

The frequency of *S. aureus* from total mastitis cases was 41.5%. This finding is similar to the 46.2% reported previously in China^45^, 41% in France^46^, and 47.2% in Italy^47^. However, higher isolation rates of *S. aureus* from milk samples were recorded in India (79.71%^48^) and Bangladesh (100%^49^).

Non-*aureus* staphylococci and mammaliicocci (NASM) are the most frequently isolated bacterial group from bovine milk samples, comprising a heterogeneous group of numerous species^50^. Recently, NAS are reclassified into CoNS including *S. lugdunensis*, and mammaliicocci including *M. sciuri* and *M. lentus*^4^. They are commonly associated with subclinical mastitis rather than clinical mastitis^51,52^. In this study, the frequency of NAS isolates in milk samples was 58.5% mainly from subclinical mastitis of both cattle and buffaloes, including *M. lentus*, *M. sciuri*, and CoNS *S. lugdunensis*. Similarly, Freu et al.^53^ reported that *M. sciuri* (3.75%), *S. lugdunensis* (0.05%), and *M. lentus* (0.01%) were the most frequent NASM species isolated from bovine clinical mastitis cases.

MRSA frequency in our study was 100%, while in China was varied from 15.5 to 47.6%^45,54^, in Korea^55 ^was 13.9%, while in Tunisia was 6.6%^56^and 48.7% in India^57^. Elfaramawy et al.^58^ reported that 67.4% of *S. aureus* bovine mastitis isolates in Egypt were MRSA and showed resistance to several antimicrobials classes. These variations in frequencies may be due to differences in sample sizes, seasons, and geographical factors^56^.

MRSA and VRSA are particularly concerning because they are often resistant to multiple antibiotics, making them difficult to treat. This can lead to serious infections that are difficult to manage and can be life-threatening^6,10^. Therfore, the increasing prevalence of these resistant strains, including MDR, XDR, and PDR phenotypes is a major public health concern. For instance, among the 27 MRSA isolates in this study, a significant number were categorized into these resistant phenotypes. PCR verified the presence of the *mec*A gene in all detected MRSA strains. This aligns with the findings of Ito et al.^59^who found that most MRSA isolates have an MDR phenotype and carry multiple resistance determinants. Glycopeptide antibiotics, including vancomycin, were frequently utilized as a treatment option for MRSA infections after the emergence of MRSA strains. However, VRSA has emerged^10^. The excessive usage of the vancomycin derivative (avoparcin) as a growth promoter or antibiotic use in food-producing animals may contribute to decreased vancomycin susceptibility^60^.

In the current study, all VRSA isolates were MRSA. This aligns with Cong et al.^10^ who reported that that pre-existing MRSA infection is typically the source of *S. aureus* isolates that have lower vancomycin susceptibility. It is worth mentioning that 85.2% of *S. aureus* isolates from milk samples were resistant to vancomycin (MIC ≥ 64 µg/mL) which represents a significant public health hazard since milk can serve as a vehicle for the acquisition of vancomycin resistance to humans.

We detected *van*A gene in 60.8% of VRSA strains, that was nearly similar to those reported by Shady et al.^61^ who detected *van*A gene in 50% of VRSA strains. However, other studies reported different percentages (86% and 27%) of *van*A gene in VRSA strains^62,63^. Additionally, 74% of VRSA strains harbored *van*B gene, which is higher than the 36% reported by Shindia et al.^63^. Only 10 VRSA isolates harbored both *van*A and *van*B genes (MIC ≥ 1024 µg/mL). Two isolates tested negative for *van* genes despite displaying high vancomycin MIC values of 64 and 128 µg/mL. The lack of *van*A/B genes in these isolates does not revoke VRSA status^64^. According to the hypothesis that these isolates’ development of vancomycin resistance is caused by thicker cell walls, the dense accumulation of vancomycin molecules within the thicker cell wall considerably slows down the inhibition of cell wall synthesis by impeding vancomycin molecules’ ability to efficiently pass through the thicker cell-wall layers^65^. Previous studies have recorded the emergence of VRSA in clinical samples without the presence of *van* genes^66^.

Beyond being resistant to antimicrobial agents, MRSA isolates produce biofilms, which help them survive in the infection niche^67^. All 27 MRSA isolates in this study produced biofilm, categorized as strong (55.6%) and moderate (44.4%) biofilm producers. Similarly, all 23 VRSA isolates produced biofilm, with 14 strong and 9 moderate producers. Phenotypic resistance and biofilm formation were significantly correlated, with vancomycin resistance being enhanced by the biofilm microenvironment^68^. Of the isolates that produced biofilm, 74% had biofilm genes found in them; *ica*A and *ica*D were equally prevalent, and just one isolate had no genes. Our results are in line with previous studies^69^ that identified the *ica* genes in all isolate, particularly the *ica*A and *ica*D genes^70^. However, 75.3% of isolates have *ica* genes^71^, with a lesser percentage having both *ica*A and *ica*D.

Staphylococcal enterotoxin genes (*sea*,* seb*,* sec*,* sed*, and *see*) have been detected in 46.9% of *S. aureus* isolated from bovine mastitis^72^. These enterotoxins are stable at high temperatures and retain their biological activity in milk even after pasteurization^15^. The *sea* gene was the most prevalent (59.3%), followed by *seb* gene (40.7%), *sed* gene (33.3%), *sec* gene (18.5%), and *see* gene (14.8%). *S. aureus* isolates from bovine mastitis harbored these enterotoxin-related genes at different percentages. Grispoldi et al.^73^ found *sea* (35.3%), *sed* (29.4%), *seb* and *sec* genes (5.9%, each) in *S. aureus* isolates. Moreover, Algammal et al.^42^ reported that 30% and 10% of isolates harbored *sea* and *sec* genes, respectively. Monistero et al.^74^ found the *sea* gene in 65.6% of isolates, and Neelam et al.^48^ found the *seb* in 9.1%, *sec* in 1.8%, and *sed* in 7.3%. This variation in the frequency of enterotoxin-related genes among *S. aureus* isolated from bovine mastitis may be linked to the geographical origin of the isolates and various clinical signs of infection. Acute infections have been reported, although most infections are chronic^13^.

Protein A is a cell wall component of *S. aureus* that hinders phagocytosis by neutrophils and the polymorphic X-region of the protein A gene (*spa*) was used for molecular typing of MRSA strains^75^. In this study, the *spa* gene (X-region) was detected in 70.4% of *S. aureus* isolates, showing remarkable polymorphism with amplicon sizes of 220 bp, 315 bp and 610 bp. Algammal et al.^42^ found 26 out of 30 MRSA strains (86.6%) were positive for the *spa* gene, showing polymorphism with amplicon sizes of 140 bp, 270 bp and 290 bp. The variations in the amplicon size of the X-region of the *spa* gene could be due to deletions of repeats and might give evidence for evolutionary changes^76^. Similarly, Kalorey et al.^77^ reported that the amplification of the polymorphic *spa* gene segment encoding the X-region was detected in 70.3% of *S. aureus* isolates from subclinical mastitis in India. In addition, Momtaz et al.^78^ found that 25.6% of isolates from bovine clinical and subclinical mastitis in Iran contained the X-region of the protein A gene. *spa* gene was not found in all isolates because *spa* typing can be unreliable for *S. aureus* strains with mutations in the IgG binding domain, as these mutations can disrupt the primer binding site^79^. Additionally, some strains may lack *spa* entirely due to mutations in the *spa* gene’s 5’ untranslated region^80^. This absence of *spa* can result in increased capsule production, which paradoxically makes the bacteria more vulnerable to phagocytosis and killing by the host immune system^16^.

Eight different *spa* types were identified in this study including: t267, t037, t011, t834, t2663, t1575, t1081, and t127. MRSA strains belonged to *spa* types t267, t037, t011, t834, t2663, and t1575 (*n* = 1) and t1081 (*n*= 3). Huber et al.^81^ detected *spa* types t011 (*n* = 7), t034 (*n* = 11), and t127 (*n* = 1) in mastitis milk samples in Switzerland. The *spa* type t011 was also found in dairy cows in Germany^82^. Cvetnić et al.^83^ isolated t267 (7.33%) from milk of cows with subclinical mastitis in Croatia. The predominant *spa* type detected in this study was t127 (52.6%), which aligns with previous studies by Juhász-Kaszanyitzky et al.^84^ and Basanisi et al.^85^ which found t127 to be common in MRSA isolates from bovine mastitis in Hungary and Italy. On the other hand, Vanderhaeghen et al.^86^ reported that t011 was the most widely distributed *spa* type associated with clinical and subclinical mastitis in Belgian cows.

Two necessary gene complexes constitute a SCC*mec* element: the *mec* complex and the cassette chromosomal recombinase (ccr) complex, responsible for the movements of SCC*mec*^87,88^. SCC*mec* I, II or III are usually resistant to multiple drugs, and SCC*mec* subtypes can be classified by differences in the J regions^89^. In this study, SCC*mec* typing revealed SCC*mec* I (*n* = 17), SCC*mec* II (*n* = 6) and SCC*mec* III (*n* = 4), and were classified into four subtypes (1.1.1, 1.1.2, 1.n.1, and 4.1.1). SCC*mec* I 1.n.1 was the predominant type identified from clinical and subclinical mastitis cases on farms and backyards. MRSA isolates from different areas carried SCC*mec*, such as SCC*mec* III or SCC*mec* XII in China, SCC*mec* V in Malaysia, SCC*mec* IX in Thailand, and SCC*mec* IVb, or V in Hong Kong^17,90,91^.

## Conclusions

The emergence of MRSA and VRSA in cattle complicates the treatment of bovine mastitis caused by *S. aureus* and poses a potential risk of animal-to-human transmission. Screening milk from bovine mastitis cases and the genotypic analysis of the isolated strains are essential to estimate MRSA spread and understand transmission mechanisms.

The high prevalence of MDR and XDR isolates in the research area has been linked to inadequate infection control and preventive procedures as well as the overuse and reckless use of antibiotics in cattle husbandry. Therefore, to stop MRSA and VRSA from spreading further, both individuals and governments need to take action.

## Electronic supplementary material

Below is the link to the electronic supplementary material.

Supplementary Material 1

## Data Availability

Data supporting the findings of this study are available within the article and its supplementary materials. Sequence data that support the findings of this study have been deposited in the GenBank with accession nos: PP249546 - PP249564.
