# Supplementary material for: Vancomycin-resistant Staphylococcus aureus endangers Egyptian dairy herds
Source: Sci Rep. 2024 Dec 23;14:30606. doi: 10.1038/s41598-024-81516-6 (PMC11666717; doi:10.1038/s41598-024-81516-6)
Supplement: Supplementary file 1 — Supplementary Material 1 [file 41598_2024_81516_MOESM1_ESM.doc]

**Table 1S** : Oligonucleotide primer sequences used in PCR assay

| **Target gene** | **Primer** | **Primer sequence**  **(5'-3')** | **Amplified product (bp)** | **Reference** |
| --- | --- | --- | --- | --- |
| *Sa0836* | *S. aureus* - F | TCGAAATTAAATGTTGTCGTGTCTTC | 573 | Liu *et al.*, 2005 |
|  | *S. aureus* **-** R | TCATTTTTGACATGRAGAGAAACATC |  |  |
| 16S rRNA | STAR1 | GCGGATCCATCTATAAGTGA | 285 | Edwards *et al.,* 2001 |
|  | STAF1 | GGGTGAGTAACACGTGGA |  |  |
| *mec* A | *mec*A - 1 | GTAGAAATGACTGAACGTCCGATAA | 310 | Spanu *et al*., 2004 |
|  | *mec*A - 2 | CCAATTCCACATTGTTTCGGTCTAA |  |  |
| *van* A | *van*A - F | ATGAATAGAATAAAAGTTGC | 1,032 | Saha *et al.,* 2008 |
|  | *van*A - R | TCACCCCTTTAACGCTAATA |  |  |
| *van B* | *van*B - F | GTGACAAACCGGAGGCGAGGA | 433 | Clark et al., 1993 |
|  | *van*B - R | CCGCCATCCTCCTGCAAAAAA |  |  |
| *sea* | *SEA* - F | GGTTATCAATGTGCGGGTGG | 102 | Mehrotra *et al.*, 2000 |
|  | *SEA* - R | CGGACTTTTTTCTCTTCGG |  |  |
| *seb* | *SEB* - F | GTATGGTGGTGTAACTGAGC | 164 | Mehrotra *et al.*, 2000 |
|  | *SEB* - R | CCAAATAGTGACGAGTTAGG |  |  |
| *sec* | *SE*C - F | AGATGAAGTAGTTGATGTGTATGG | 451 | Mehrotra *et al.*, 2000 |
|  | *SE*C - R | CACACTTTTAGAATCAACCG |  |  |
| *sed* | *SED* - F | CCAATAATAGGAGAAAATAAAAG | 278 | Mehrotra  *et al*., 2000 |
|  | *SED* - R | ATGGTATTTTTTTTCGTTC |  |  |
| *see* | *SEE* - F | AGGTTTTTTCACAGGTCATCC | 209 | Mehrotra  *et al*., 2000 |
|  | *SEE* - R | CTTTTTTTTCTTCGGTCAATC |  |  |
| *ica*A | *ica*A - F | CCTAACTAACGAAAGGTAG | 1,315 | Vasudevan *et al*., 2003 |
|  | *ica*A - R | AAGATATAGCGATAAGTGC |  |  |
| *icaD* | *ica*D - F | ATGGTCAAGCCCAGACAGAG | 198 | Rohde *et al.*, 2001 |
|  | *ica*D - R | AGTATTTTCAATGTTTAAAGCAA |  |  |
| *tsst* | *tsst*-1 - F | GCTTGCGACAACTGCTACAG | 559 | Løvseth *et al.,* 2004 |
|  | *tsst*-1 - R | TGGATCCGTCATTCATTGTTAT |  |  |
| *luk*MF | *luk*M - F | AAACGCGCAGTTAATAAAAAG | 975 | Hoekstra *et* *al.,* 2018 |
|  | *luk*M - R | AGCATTAGGTCCTCTTGTCG |  |  |
| *hlb* | *Hlb* - 1 | TGCAGAAGATGGTGGCGTAG | 974 | Miruka *et al.*, 2022 |
|  | *Hlb* - 2 | CTGGGGCTATTGGTCTGGTG |  |  |
| *spa-X* | 1095- F | AGACGATCCTTCGGTGAGC | Variable | Harmsen *et al*., 2003 |
|  | 1517 -R | GCTTTTGCAATGTCATTTACTG |  |  |
| *mecA (m*A*1-m*A*2)* | mA1 | TGCTATCCACCCTCAAACAGG | 286 | Kondo *et al.*, 2007 |
|  | mA2 | AACGTTGTAACCACCCCAAGA |  |  |
| *ccrA1*-*ccrB* (_1-_c) | α 1 | AACCTATATCATCAATCAGTACGT | 695 |  |
| *ccrA2*-*ccrB* (_2-_c) | α 2 | TAAAGGCATCAATGCACAAACACT | 937 |  |
| *ccrA3*-*ccrB* (_3-_c) | α 3 | AGCTCAAAAGCAAGCAATAGAAT | 1,791 |  |
|  | β c | ATTGCCTTGATAATAGCCITCT |  |  |
| *ccrA4*-*ccrB4* (_4.2-_4.2) | α 4.2 | GTATCAATGCACCAGAACTT | 1,287 |  |
|  | β 4.2 | TTGCGACTCTCTTGGCGTTT |  |  |
| *ccrC* (_R-_F) | γR | CCTTTATAGACTGGATTATTCAAAATAT | 518 |  |
|  | γF | CGTCTATTACAAGATGTTAAGGATAAT |  |  |
| *mecA*-*mecI* (mA7-mI6) | mI6 | CATAACTTCCCATTCTGCAGATG | 1,963 |  |
| *mecA*-IS*1272* upstream of *mecA* (mA7-IS7) | IS7 | ATGCTTAATGATAGCATCCGAATG | 2,827 |  |
| *mecA*-IS*431* upstream of *mecA* (mA7-IS2 iS-2) | IS2(iS-2) | TGAGGTTATTCAGATATTTCGATGT | 804 |  |
|  | mA7 | ATATACCAAACCCGACAACTACA |  |  |
| E007 in type I.1 SCC*mec* (1a3-la4) | 1a3 | TTTAGGAGGTAATCTCCTTGATG | 154 |  |
|  | 1a4 | TTTTGCGTTTGCATCTCTACC |  |  |
| CQ02 in type IV.1 (IVa) SCC*mec* (4al-4a3) | 4al | TTTGAATGCCCTCCATGAATAAAAT | 458 |  |
|  | 4a3 | AGAAAAGATAGAAGTTCGAAAGA |  |  |
| M001 in type IV.2 (IVb) SCC*mec* (4b3-4b4) | 4b3 | AACCAACAGTGGTTACAGCTT | 726 |  |
|  | 4b4 | CGGATTTTAGACTCATCACCAT |  |  |
| CR008 in type IV.3 (IVc)  SCC*mec* (4c4-4c5) | 4c4 | AGGAAATCGATGTCATTATAA | 259 |  |
|  | 4c5 | ATCCATTTCTCAGGAGTTAG |  |  |
| CD002 in type IV.4  (IVd) SCC*mec* (4d3-4d4) | 4d3 | AATTCACCCGTACCTGAGAA | 1,242 |  |
|  | 4d4 | AGAATGTGGTTATAAGATAGCTA |  |  |
| *kdpB* in type II.1 (IIa)  SCC*mec* (kdpB1- kdpB2) | kdpB1 | GATTACTTCAGAACCAGGTCAT | 287 |  |
|  | kdpB2 | TAAACTGTGTCACACGATCCAT |  |  |
| SA01 in type II.2 (IIb) SCC*mec* (2b3-2b4) | 2b3 | GCTCTAAAAGTTGGATATGCG | 1,518 |  |
|  | 2b4 | TGGATTGAATCGACTAGAATCG |  |  |
| IIE03 in type II.3 (IIE) SCC*mec* or M001 in type IV.2 (IVb) SCC*mec* (4b3-4b4) | 4b3 | AACCAACAGTGGTTACAGCTT | 726 |  |
|  | 4b4 | CGGATTTTAGACTCATCACCAT |  |  |
| RN06 in type II.4 SCC*mec* (II4-3-II4-1) | II4-3 | GTACCGCTGAATATTGATAGTGAT | 2,003 |  |
|  | II4-1 | ACTCTAATCCTAATCACCGAAC |  |  |
| Z004 in type III.1 SCC*mec* (3a1-3a2) | 3a1 | ATGGCTTCAGCATCAATGAG | 503 |  |
|  | 3a2 | ATATCCTTCAAGCGCGTTTC |  |  |
| V024 in type V SCC*mec* (5a1-a2) | 5a1 | ACCTACAGCCATTGCATTATG | 1,159 |  |
|  | 5a2 | TGTATACATTTCGCCACTAGCT |  |  |
| *ermA*-CN030 or CZ021 in J2 region of type II.1 (IIa) or type III.1 SCC*mec* (ermA1-mN5) | ermA1 | TGAAACAATTTGTAACTATTGA | 2,756 |  |
| *cadB*-CN030 or CZ021 in  J2 region of type II.1 (IIa) or type III.1 SCC*mec* (cad4-mN5) | cad4 | ATTGCGATTCTTTCCGATATGG | 1,540 |  |
|  | mN5 | TTGCTTCGGGACTTACCTCTAGT |  |  |
| *mecA*-*ant*(*4*_) in pUB110 (mA1-ant1) | ant1 | CAGACCAATCAACATGGCACC | 4,952 |  |
| *mecA*-*tetK* in pT181 (mA1-pT181-2) | pT181-2 | AGGTTTATTGTCACTACAATTGA | 7,406 |  |
|  | mA1 | TGCTATCCACCCTCAAACAGG |  |  |
| *pls* (CE010) in type I SCC*mec* (1a1-1a2) | 1a1 | ATTCCATATGAAACTAAACGCGT | 1,065 |  |
|  | 1a2 | TAGTGAACCAAATAATGTGCCATT |  |  |
| *mer* operon (merA2-merG) | merA2 | TCTTCACAGCCTGTGCATGTCATGCCT | 1,546 |  |
|  | merG | TGATACCGCGAATGAATCAAAGGT |  |  |
| J region in SCC*mercury* (mN21-mN22) | mN21 | TCATCTTTAACTACGATGGTGT | 577 |  |
|  | mN22 | ACTACAGCCATCTTCAGATAGA |  |  |
| *orfX*-*mecA* (cR1-mA3) | cR1 | AAGAATTGAACCAACGCATGA | 11,756b |  |
|  | mA3 | AACGTTACAAGATATGAAGTGGTAAATGGTA |  |  |
| *mecA*-*ermA* (mA2-ermA1) | mA2 | AACGTTGTAACCACCCCAAG | 11,020b |  |
|  | ermA1 | TGAAACAATTTGTAACTATTGA |  |  |
| *ermA-ccr* gene complex (ermA3-2AJ1) | ermA3 | TGGGTAAACCGTGAATATCGTGT | 9,937b |  |
|  | 2AJ1 | ATTAGCCGATTTGGTAATTGA |  |  |
| *ccr* gene complex chromosomal region flanking SCC*mec* (c-cL4) | β c | ATTGCCTTGATAATAGCCITCT | 15,000c |  |
|  | cL4 | CAGTCGCATCAAATGTCTCTAATG |  |  |

**Table 2S:** Antibiograms, vancomycin MIC, resistance genes, biofilm formation ability, and virulence factors of 27 MRSA isolates

| **Antibiotype**  **No.** | **Isolates code** | **No. of**  **isolates/**  **pattern** | **No. of**  **resistant**  **antimicrobials** | **Resistance phenotype** | **Resistance pattern** | **MAR**  **index** | **Vancomycin**  **MIC**  **(µg/mL)** | **Vancomycin**  **resistance**  **genes** | **Biofilm-forming**  **ability** | | **Biofilm**  **genes** | **Virulence genes** | ***spa* type** | **GenBank accession no.** | **SCCmec type** | **Subtype** |
| --- | --- | --- | --- | --- | --- | --- | --- | --- | --- | --- | --- | --- | --- | --- | --- | --- |
|  |  |  |  |  |  |  |  |  | OD  570 + SD* | Degree |  |  |  |  |  |  |
| **A1** | 4  6  23 | 3 | 33 | PDR | AK, AM, AMC, B, C, CAZ, CE, CIP, CN, CPT, CXM, DA, DAP, DO, E, F, FA, FOS, LNZ, ME, NET, NOR, OX, P, QD, RA, SAM, SP, SXT, TE, TGC, TZP, VA | 1 | 64  1024  1024 | ND  *Van*A, *Van*B  *Van*A, *Van*B | 0.686+ 0.003  0.910+ 0.001  0.603+ 0.001 | Strong  Strong  Strong | *ica*D  *ica*A,  *ica*D  *ica*A,  *ica*D | *hlb, LukMF,spa*  *hlb, LukMF, tsst*, *spa, seb, sec, sed*  *hlb, LukMF, tsst*, *seb, sed, see* | t127  t011 | PP249549  PP249551 | II  II  I | 1.1.1  4.1.1  1.n.1 |
| **A2** | 5 | 1 | 27 | MDR | AK, AM, AMC, B, C, CAZ, CE, CIP, CN, DA, DAP, DO, E, FOS, ME, NET, OX, P, QD, RA, SAM, SP, SXT, TGC, TE, TZP, VA | 0.82 | 128 | ND | 0.257+ 0.003 | Moderate | *ica*D | *hlb, LukMF*  *tsst*, *spa,sea, sed* | t037 | PP249550 | II | 1.1.1 |
| **A3** | 13 | 1 | 27 | MDR | AM, AMC, B, C, CAZ, CE, CIP, CXM, DA, DAP, DO, E, FA, FOS, ME, NET, NOR, OX, P, RA, SAM, SP, SXT, TE, TGC, TZP, VA | 0.82 | 1024 | *Van*A, *Van*B | 0.688+ 0.001 | Strong | *ica*A,  *ica*D | *hlb, LukMF*  *tsst*, *spa, sea* | t127 | PP249558 | I | 1.n.1 |
| **A4** | 15 | 1 | 27 | XDR | AM, AMC, B, C, CAZ, CE, CIP, CN, DA, DAP, DO, E, FA, FOS, ME, NET, NOR, OX, P, QD, RA, SAM, SP, SXT, TGC, TZP, VA | 0.82 | 1024 | *Van*B | 0.310+ 0.001 | Strong | *ica*A,  *ica*D | *hlb, LukMF*  *spa, sec, see* | t1081 | PP249560 | III | 1.1.1 |
| **A5** | 18 | 1 | 27 | MDR | AK, AM, AMC, B, C, CAZ, CE, CIP, CN, CXM, DA, DAP, DO, E, FOS, ME, NET, NOR, OX, P, RA, SAM, SP, SXT, TE, TGC, VA | 0.82 | 1024 | *Van*A, *Van*B | 0.326+ 0.001 | Strong | *ica*A,  *ica*D | *hlb, LukMF*  *sea* |  |  | I | 1.n.1 |
| **A6** | 20 | 1 | 27 | XDR | AM, AMC, B, C, CAZ, CE, CIP, CN, DA, DAP, DO, E, FA, FOS, ME, NET, NOR, OX, P, QD, RA, SAM, SP, SXT, TGC, TE, VA | 0.82 | 64 | *Van*B | 0.714+ 0.001 | Strong | *ica*D | *hlb, LukMF, tsst* |  |  | I | 1.n.1 |
| **A7** | 7 | 1 | 26 | MDR | AM, AMC, B, C, CAZ, CE, CIP, CN, CXM, DAP, DO, E, F, FA, FOS, ME, NET, NOR, OX, P, SAM, SP, SXT, TGC, TZP, VA | 0.79 | 1024 | *Van*A | 0.614+ 0.001 | Strong | *ica*A, *ica*D | *hlb, LukMF*  *tsst*, *spa, sea, sed* | t843 | PP249552 | I | 1.n.1 |
| **A8** | 9 | 1 | 26 | MDR | AM, AMC, B, CAZ, CE, CIP, CN, CXM, DA, DAP, DO, E, FOS, ME, NET, NOR, OX, P, QD, SAM, SP, SXT, TGC, TE, TZP, VA | 0.79 | 1024 | *Van*A, *Van*B | 0.597+ 0.001 | Strong | *ica*A, *ica*D | *hlb,LukMF*  *tsst*, *spa, sea, sed* | t1081 | PP249554 | I | 1.n.1 |
| **A9** | 11 | 1 | 26 | MDR | AM, AMC, B, C, CAZ, CE, CN, CXM, DA ,DAP, DO, E, FA, FOS, ME, NET, OX, P, RA, SAM, SP, SXT, TGC, TE, TZP, VA | 0.79 | 64 | *Van*A, *Van*B | 0.201+ 0.001 | Moderate | *ica*A | *hlb, LukMF*  *spa, sea, seb* | t127 | PP249556 | I | 1.n.1 |
| **A10** | 17 | 1 | 26 | XDR | AM, AMC, B, CAZ, CE ,CN, CXM, DA ,DAP, DO, E, FA, FOS, ME, NET, OX, P, QD, RA, SAM, SP, SXT, TGC, TE, TZP, VA | 0.79 | 1024 | *Van*A, *Van*B | 0.204+ 0.001 | Moderate | *ica*A,  *ica*D | *hlb, LukMF*  *sea, seb, sed* |  |  | III | 1.1.2 |
| **A11** | 24 | 1 | 26 | MDR | AK, AM, AMC, B, C, CAZ, CE, CIP, CN, DA, DAP, E, FOS, ME, NET, NOR, OX, P, RA, SAM, SP, SXT, TE, TGC, TZP, VA | 0.79 | 512 | *Van*B | 0.476+ 0.001 | Strong | *ica*A, *ica*D | *hlb,LukMF*  *spa, seb*, see | t127 | PP249564 | I | 1.n.1 |
| **A12** | 10 | 1 | 25 | MDR | AM, AMC, B, CAZ, CE, CN, CXM, DA, DAP, DO, E, FA, FOS, ME, NET, OX, P, QD, SAM, SP, SXT, TGC, TE, TZP, VA | 0.76 | 64 | *Van*B | 0.193+ 0.006 | Moderate | *ica*A | *Hlb ,LukMF*  *spa, sea* | t2663 | PP249555 | I | 1.n.1 |
| **A13** | 12 | 1 | 25 | MDR | AM, AMC, B, CAZ, CE, CIP, CN, CXM, DAP, DO, E, FA, FOS, ME, NET, OX, P, RA , SAM, SP, SXT, TGC, TE, TZP, VA | 0.76 | 64 | *Van*B | 0.276+ 0.002 | Moderate | *ica*A | *hlb,LukMF*  *tsst*, *spa, sea,*  *seb, sed* | t1575 | PP249557 | I | 1.n.1 |
| **A14** | 21 | 1 | 25 | XDR | AK, AM, AMC, B, CAZ, CE, CN, DA ,DAP, DO, E, FA, FOS, ME, NET, OX, P, QD, RA, SAM, SP,SXT, TGC, TE, VA | 0.76 | 64 | *Van*A, *Van*B | 0.242+ 0.002 | Moderate | *ica*A | *hlb, LukMF*  *sea* |  |  | I | 1.n.1 |
| **A15** | 25 | 1 | 25 | MDR | AK, AM, AMC, B, CAZ, CE, CIP, CN, DA, DAP, DO, E, ME, NET, NOR, OX, P, RA, SAM, SP, SXT, TGC, TE, TZP, VA | 0.76 | 512 | *Van*A, *Van*B | 0.287+ 0.006 | Strong | *ica*A, *ica*D | *hlb, LukMF, sea, seb, sec, see* |  |  | I | 1.n.1 |
| **A16** | 27 | 1 | 25 | MDR | AM, AMC, B, CAZ, CE, CIP, CN, CXM, DA, DAP, DO, E, FA, FOS, ME, NET, OX, P, RA, SAM, SP, SXT, TGC, TZP, VA | 0.76 | 128 | *Van*B | 0.192+ 0.007 | Moderate | ND | *hlb, LukMF* |  |  | I | 1.n.1 |
| **A17** | 2 | 1 | 24 | MDR | AM, AMC, B, C, CAZ, CE, CN, CXM, DA, DAP, DO, E, FA, FOS, ME, NET, OX, P, SAM, SP, SXT, TGC, TZP, VA | 0.73 | 64 | *Van*B | 0.201+ 0.017 | Moderate | *ica*A | *hlb, LukMF*  *tsst*, *spa, sea, seb, sed* | t127 | PP249547 | II | 1.1.1 |
| **A18** | 3 | 1 | 24 | MDR | AM, AMC, B, CAZ, CE, CN, CXM, DA, DAP, DO, E, FA, FOS, ME, NET, OX, QD, SAM, SP, SXT, TGC, TE, TZP, VA | 0.73 | 256 | *Van*A, *Van*B | 0.307+ 0.003 | Strong | *ica*A, *ica*D | *hlb, LukMF, spa* | t267 | PP249548 | II | 1.1.1 |
| **A19** | 14 | 1 | 24 | MDR | AM, AMC, B, C, CAZ, CE, CN, DA, DAP, DO, E, FA, FOS, ME, NET, OX, P, RA, SAM, SP, SXT, TGC, TZP, VA | 0.73 | 1024 | *Van*A | 0.437+ 0.002 | Strong | *ica*A, *ica*D | *hlb, LukMF*  *tsst*, *spa, sea, sec* | t127 | PP249559 | III | 1.1.1 |
| **A20** | 1 | 1 | 23 | MDR | AM, AMC, B, CAZ, CE, CN, CXM, DA, DAP, DO, E, FA, ME, NET, OX, P, RA, SAM, SP, SXT, TGC, TZP, VA | 0.7 | 64 | *Van*A | 0.192+ 0.027 | Moderate | *ica*A, | *hlb, LukMF*  *spa sea, seb, sed* | t127 | PP249546 | II | 1.1.1 |
| **A21** | 19 | 1 | 23 | MDR | AM, AMC, B, CAZ, CE, CIP, DA, DAP, DO, E, FA, ME, NET, NOR, OX, P, RA, SAM, SP, SXT, TGC, TE, TZP | 0.7 | - |  | 0.564+ 0.005 | Strong | *ica*D | *hlb,LukMF tsst*,  *spa* | t127 | PP249562 | I | 1.n.1 |
| **A22** | 22 | 1 | 22 | MDR | AM, AMC, B, C, CAZ, CE, CIP, DA, DAP, DO, E, FA, FOS, ME, NET, OX, P, SAM, SP, TGC, TZP, VA | 0.67 | 1024 | *Van*A | 0.597+ 0.002 | Strong | *ica*A, *ica*D | *hlb,LukMF*  *tsst*, *spa,sea, seb,*  *sec* | t1081 | PP249563 | I | 1.n.1 |
| **A23** | 26 | 1 | 22 | MDR | AM, AMC, B, CAZ, CE, CIP, CN, CXM, DA, DAP, E, FA, ME, NET, NOR, OX, P, RA, SAM, SXT,TGC, TE | 0.67 | - |  | 0.230+ 0.000 | Moderate | *ica*A, *ica*D | *hlb, LukMF* |  |  | I | 1.n.1 |
| **A24** | 16 |  | 21 | MDR | AM, B, CAZ, CE, CN, CXM, DA, DAP, DO, E, F, ME, NET, OX, P, QD, SAM, SP, TGC, TE, TZP | 0.64 | - |  | 0.186+ 0.005 | Moderate | *ica*D | *hlb,LukMF*  *spa, sea, seb* | t127 | PP249561 | III | 1.1.2 |
| **A25** | 8 |  | 20 | MDR | AM, B, C, CAZ, CE, CIP, CN, CXM, DA, DAP, DO, E, F, ME, NET, NOR, OX, SAM, SP, TGC | 0.61 | - |  | 0.214+ 0.001 | Moderate | *ica*D | *hlb, LukMF*  *spa* | t127 | PP249553 | I | 1.n.1 |

AK, amikacin; AM, ampicillin; AMC, amoxicillin/ clauvalanic acid; B, bacitracin; C, chloroamphenicol; CAZ, ceftazidime; CE, cephradine; CIP, ciprofloxacin; CN, gentamycin; CPT, ceftarolline; CXM, cefuroxime; DA, clindamycin; DAP, daptomycin; DO, doxycycline; E, erythromycin; F, nitrofurantoin; FD, fusidic acid; FOS, :fosfomycin; LNZ, linezolid; ME, methicillin; NET, neitilmicin; NOR, norfloxacin; OX, oxicillin; P, penicillin; QD, quinopristin/ dalfopristin; RA, rifampin; SAM, ampicillin+sulbctam; SP, spiramycin; SXT, trimethoprim/Sulfamethoxazole; TE, tetracycline; TGC, tigecycline; TZP, pipracillin+tazobactam; VA, vancomycin. No., number, ND, not detected, *Average optical density value ± standard deviations (SD). Biofilm genes were assigned for biofilm-producing isolates.
